# Predictors of functional mitral regurgitation recurrence after percutaneous mitral valve repair

**DOI:** 10.1007/s00380-021-01828-9

**Published:** 2021-04-03

**Authors:** Katharina Hellhammer, Jean M. Haurand, Maximilian Spieker, Peter Luedike, Tienush Rassaf, Tobias Zeus, Malte Kelm, Ralf Westenfeld, Patrick Horn

**Affiliations:** 1 Division of Cardiology, Pulmonology, and Vascular Medicine, Medical Faculty, Heinrich-Heine University, University Hospital Düsseldorf, Moorenstrasse 5, 40225 Düsseldorf, Germany; 2 West German Heart and Vascular Center Essen, Department of Cardiology and Vascular Medicine, University Hospital Essen, Essen, Germany; 3 Cardiovascular Research Institute, Medical Faculty, Heinrich-Heine University, Düsseldorf, Germany

**Keywords:** Functional mitral regurgitation, Percutaneous mitral valve repair, Sphericity index, Heart failure

## Abstract

We aimed to identify predictors of mitral regurgitation recurrence (MR) after percutaneous mitral valve repair (PMVR) in patients with functional mitral regurgitation (FMR). Patients with FMR were enrolled who underwent PMVR using the MitraClip^®^ device. Procedural success was defined as reduction of MR of at least one grade to MR grade ≤ 2 + assessed at discharge. Recurrence of MR was defined as MR grade 3 + or worse at one year after initially successful PMVR. A total of 306 patients with FMR underwent PMVR procedure. In 279 out of 306 patients (91.2%), PMVR was successfully performed with MR grade ≤ 2 + at discharge. In 11.4% of these patients, MR recurrence of initial successful PMVR after 1 year was observed. Recurrence of MR was associated with a higher rate of heart failure rehospitalization during the 12 months follow-up (52.0% vs. 30.3%; *p* = 0.029), and less improvement in New York Heart Association (NYHA) functional class [68% vs. 19% of the patients presenting with NYHA functional class III or IV one year after PMVR when compared to patients without recurrence (*p* = 0.001)]. Patients with MR recurrence were characterized by a higher left ventricular sphericity index {0.69 [Interquartile range (IQR) 0.64, 0.74] vs. 0.65 (IQR 0.58, 0.70), *p* = 0.003}, a larger left atrium volume [118 (IQR 96, 143) ml vs. 102 (IQR 84, 123) ml, *p* = 0.019], a larger tenting height 10 (IQR 9, 13) mm vs. 8 (IQR 7, 11) mm (*p* = 0.047), and a larger mitral valve annulus [41 (IQR 38, 43) mm vs. 39 (IQR 36, 40) mm, *p* = 0.015] when compared to patients with durable optimal long-term results. In a multivariate regression model, the left ventricular sphericity index [Odds Ratio (OR) 1.120, 95% Confidence Interval (CI) 1.039–1.413, *p* = 0.003)], tenting height (OR 1.207, 95% CI 1.031–1.413, *p* = 0.019), and left atrium enlargement (OR 1.018, 95% CI 1.000–1.038, *p* = 0.047) were predictors for MR recurrence after 1 year. In patients with FMR, baseline parameters of advanced heart failure such as spherical ventricle, tenting height and a large left atrium might indicate risk of recurrent MR one year after PMVR.

## Introduction

Clinically relevant functional mitral regurgitation (FMR) is observed in up to 56% of the patients with heart failure and is associated with a poor prognosis [1]. The etiology and pathology of FMR differs from degenerative mitral regurgitation because the former is a disease of the left ventricle. FMR is related to ischemic or non-ischemic left ventricular remodeling with papillary muscle dislocation, changes in left ventricular diameter, annulus dilatation, and reduced left ventricular function. These changes in ventricular and atrial geometry lead to coaptation failure of the mitral leaflets [2]. Up to 30% of patients who undergo surgical mitral valve repair experience recurrence of mitral regurgitation (MR) within 1 year [3]. Valvular and ventricular parameters and procedure-related technical factors have been identified as predictors of recurrent MR after surgical repair [4].

Percutaneous mitral valve repair (PMVR) has emerged as an effective therapeutic option in patients with clinically relevant MR who are at high risk for mitral valve surgery. However, recurrence of MR over the long term has also been observed and is associated with a poor outcome [5, 6]. Previous studies revealed ambivalent results regarding outcome benefits after PMVR, indicating that careful patient selection is crucial [7, 8]. However, little is known about the clinical, valvular or ventricular parameters that may impact the post-interventional course and recurrence of MR after PMVR. Here, we aimed to identify pre-procedural patient characteristics and echocardiographic parameters that serve to predict the recurrence of MR within one year after PMVR in patients with FMR.

## Methods

### Study population

Altogether, 306 consecutive patients were enrolled who underwent PMVR for clinically significant FMR with the MitraClip^®^ system (Abbott Vascular GmbH, Santa Clara, California, USA) between August 2010 and May 2019. All patients were considered to be at high surgical risk by an interdisciplinary heart team. In addition, all patients provided written informed consent for data acquisition and analysis. This study was approved by the local Ethics Committee (approval number 4497R) of the University of Düsseldorf and was conducted in accordance with the principles of the Declaration of Helsinki. All data were registered at www.clinicaltrials.gov (NCT02033811).

Assessment of MR followed current guidelines [9]. Patients with symptomatic severe or moderate MR with optimal medical treatment based on accepted guidelines were considered for MR treatment [10]. MR was evaluated pre-procedural and post-procedural (at discharge) by assessing the colour flow regurgitant jet, measurement of vena contracta, effective regurgitant orifice area, and regurgitation volume using transthoracic echocardiography. MR was graded as mild MR (1 +), moderate MR (2 +), moderate-to-severe MR (3 +) and severe MR (4 +) according to the current recommendation of the American Society of Echocardiography [9].

The left ventricular sphericity index was calculated as the ratio between the larger cross-sectional diameter and the larger longitudinal diameter of the left ventricle in end-diastolic apical four-chamber view. Leaflet tethering was assessed by the tenting area (area between annulus plane and mitral valve leaflets in early systole) and the tenting height (the distance from the annulus plane of the mitral valve to the leaflet coaptation point). Individual leaflet tethering was evaluated by measuring the tethering angle of the respective leaflet.

### Percutaneous mitral valve repair

Technical details of the MitraClip^®^ system and procedure have been previously described. The procedure was performed under deep sedation or general anesthesia and guided by transesophageal echocardiography as described previously [11].

Procedural success was defined as implantation of one or more Clips leading to a reduction of MR of at least one grade to MR grade ≤ 2 + , assessed at discharge transthoracic echocardiography.

Recurrence of MR was defined as MR grade 3 + or worse at one year after initially successful PMVR.

The definition of major adverse cardiac and cerebrovascular events (MACCEs) included in-hospital death, myocardial infarction, and stroke. Minor vascular complications were defined as minor vascular and minor bleeding complication according to the Mitral Valve Academic Research Consortium (MVARC) [12]. A major vascular complication was defined according to MVARC as overt bleeding either associated with a drop in the haemoglobin level of at least 3.0 g/dl or requiring transfusion of three units of whole blood, or causing permanent injury or requiring surgery. Acute kidney failure was defined according to the Acute Kidney Injury Network definition [12].

### Statistical analysis

Pre-procedural echocardiographic parameters, demographic data and follow-up data (1 year after procedure) were analyzed. Continuous variables were tested for normal distribution with the Kolmogorov–Smirnov test and were reported as median (interquartile range). In case of a normal distribution, Student`s unpaired t-test was performed to compare the means between the two groups. Continuous variables not following a normal distribution were compared using the Mann–Whitney *U* Test. For categorical variables, frequency in percentage was reported. Categorical variables were evaluated as a percentage and compared with the chi-square test or Fisher`s exact test. Univariate and multivariate logistic regression analysis was used to identify clinical and echocardiographic predictors for MR recurrence. Candidate variables for the multivariable model were those with a *p* value < 0.1 in the univariate analysis. A ROC analysis was performed to detect cut-off values.

A two-tailed *p* value < 0.05 was considered to be significant for all tests. All analyses were performed using SPSS for Windows (SPSS statistic, Version 22.0, SPSS Inc., Chicago, Illinois, USA).

## Results

From August 2010 to May 2019, 306 patients with FMR underwent PMVR at the Heart Center Düsseldorf. The MitraClip^®^ device implantation rate was 96.7% (296 patients) including 110 patients (35.9%) with 1 device implanted, 172 patients (56.2%) with 2 devices implanted, and 14 patients (4.8%) with 3 or more MitraClip^®^ devices implanted. In 249 patients, the 1° and 2° generation of MitraClip^®^ device was used, and in 47 patients, the MitraClip^®^ NTR/XTR generation was used (30/17 patients with MitraClip^®^ NTR/XTR).

**Fig. 1:**
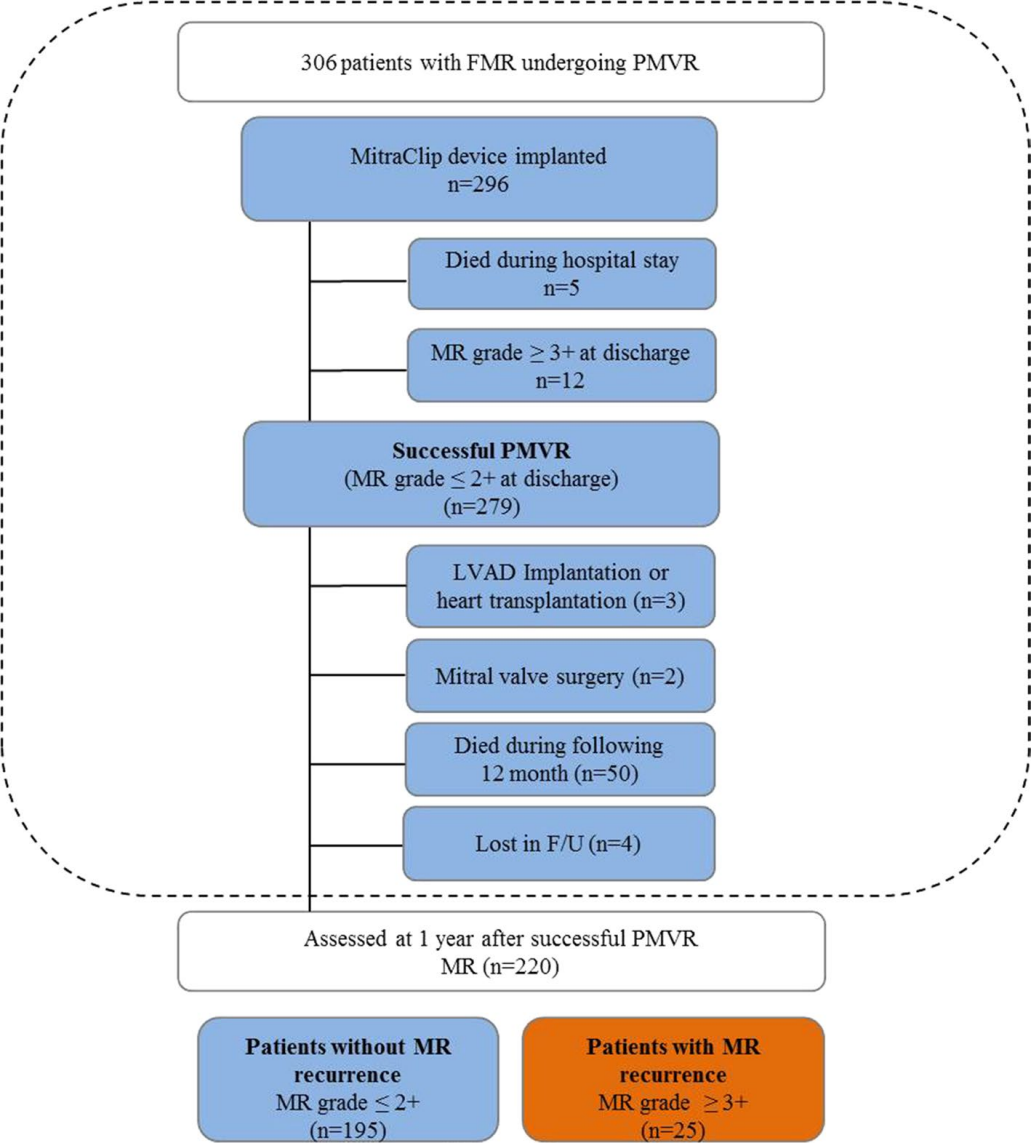
Consort Flow Diagram. *FMR* functional mitral regurgitation, *PMVR* Percutaneous mitral valve repair, *F/U* follow-up, *LVAD* left ventricular assist device

Ten patients had no device implanted because of the inability to grasp leaflets (*n* = 4), an inability to adequately reduce MR (*n* = 4), and an inadequate mitral valve orifice area (*n* = 2) (Fig. 1).

There was no patient who died during the procedure. A MACCE was reported in ten patients (3.3%) during the first 30 days post-procedural. Seven out of 306 patients undergoing PMVR died intra-hospital during the first 30 days (2.3%), five patients after device implantation, two patients in which no device could be implanted. Two patients suffered from stroke (0.7%) and one patient from acute myocardial infarction (3%). As vascular complications, minor bleedings occurred in 24 patients (7.8%), and major bleedings in 9 patients (2.9%). The bleeding complications mainly occurred at the access site that was closed by z-shaped suture and could be all successfully managed by manual compression. In two patients, vascular surgery was required post-procedural due to arterial complications caused by the sheat of invasive arterial blood pressure monitoring. Thirty-five patients (11.4%) suffered from acute kidney injury post-procedural (25 patients from acute kidney injury stage 1, nine patients from stage 2, and one patient from stage 3).

At discharge, 12 patients had MR grade 3 + or worse after MitraClip^®^ implantation. In three of this 12 patients, single leaflet detachment occurred that has led to an early worsening of the MR after the procedure. In four of these 12 patients, early MR worsening was caused by the intraprocedural damage of chordae. In five patients, no reason for the MR worsening could be identified. Two patients did not tolerate the acute worsening of MR after the procedure and underwent urgent mitral valve replacement before discharge.

Finally, in 279 out of all 306 patients (91.2%), PMVR was successfully performed with MR grade ≤ 2 + at discharge (Fig. 1).

After successful PMVR, three patients (1.1%) were bridged to heart transplantation or the implantation of a left ventricular assist device in the following 12 months. Two patients (0.7%) underwent mitral valve surgery during the following 12 months (one patient suffered from late clip detachment with recurrent MR, one patient from significant mitral stenosis post PMVR). Four patients (1.4%) were lost to follow-up. Fifty patients (17.9%) died during the following 12 months after successful PMVR (Fig. 1). Altogether, 220 patients were assessed for clinical and echocardiographic evaluation one year after initial successful PMVR.

### Efficacy of PMVR in patients with FMR

**Fig. 2:**
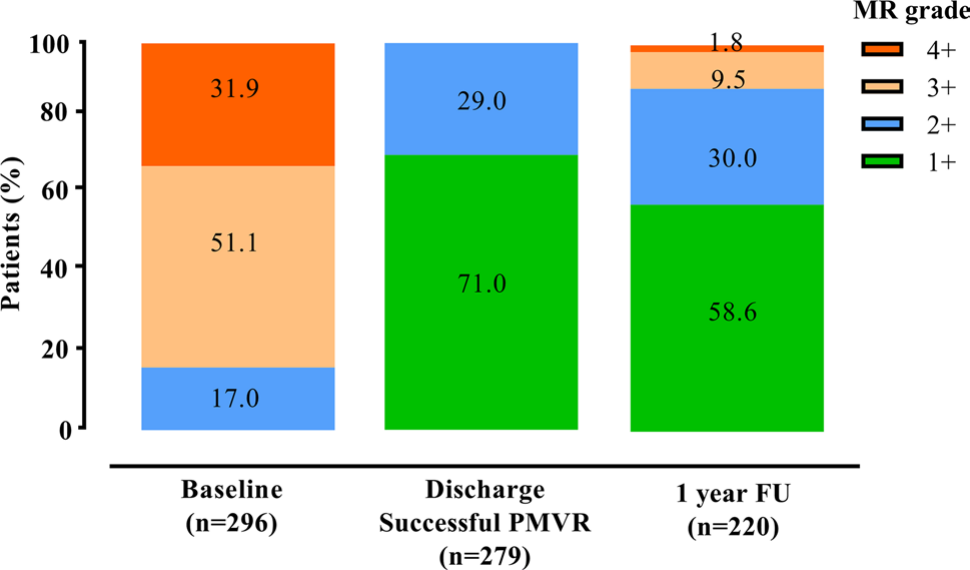
Severity of Mitral regurgitation (MR) in patients with FMR undergoing PMVR. Grade of MR severity at baseline, before discharge (post-procedural) and at 1 year follow-up. MR was graded as mild MR (1 +), moderate MR (2 +), moderate-to-severe MR (3 +) and severe MR (4 +). *MR* mitral regurgitation, *PMVR* Percutaneous mitral valve repair

After 12 months, echocardiographic evaluation showed that in four patients MR deteriorated from MR grade 2 to grade 4. In nine-teen patients, MR deteriorated from grade 2 at discharge to grade 3 at 1 year follow-up, and in 2 patients MR deteriorated from grade 1 + to grade 3 (Fig. 2). Taken together, 1-year after initial successful PMVR, 25 out of the 220 patients (11.4%) who survived had recurrent MR grade 3 + or worse. Accordingly, 195 of the 220 survived patients (88.6%) had MR grade 1 + or 2 + at 1 year follow-up.

### Clinical outcome of recurrent MR after PMVR in FMR

**Fig. 3:**
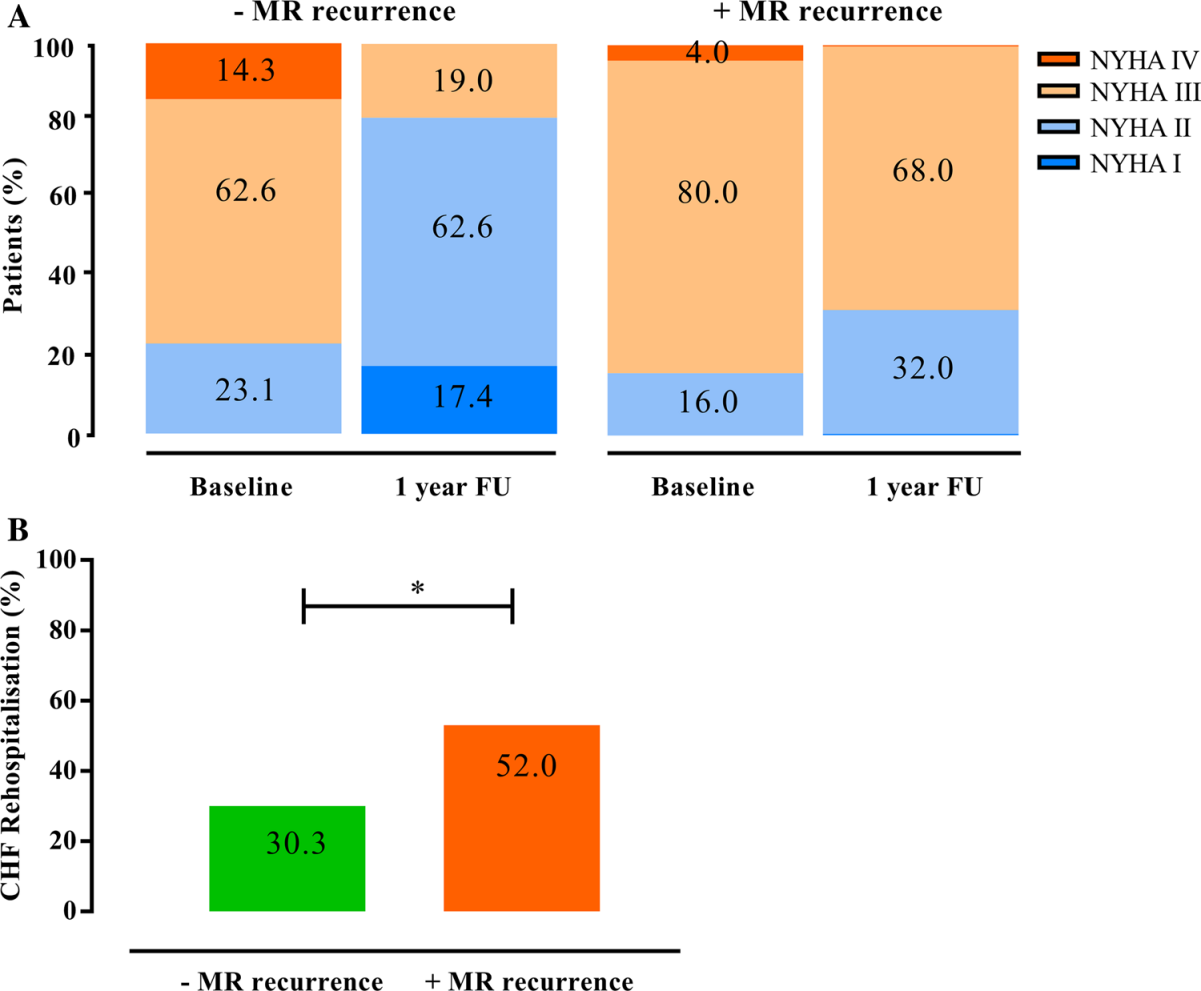
Clinical outcome of patients with FMR undergoing PMVR. **a** NYHA functional class at baseline and at 1 year after PMVR. **b** Rate of heart failure rehospitalisation during the first year after PMVR. *Indicates *p* < 0.05 between the groups. *FMR* functional mitral regurgitation, *NYHA* New York Heart Association, *PMVR* Percutaneous mitral valve repair

At baseline, 76.9% of the patients without MR recurrence presented with severe clinical symptoms with New York Heart Association (NYHA) functional class III or IV (Fig. 3a). One-year after PMVR, clinical symptoms improved as 19.0% of the patients presented with NYHA functional class III or IV (*p* = 0.001). In the MR recurrence group, clinical symptoms did not improve (84.0% of patients presented with NYHA functional class III or IV at baseline and 68% of the patients one year after PMVR, *p* = 0.185).

The rate of heart failure rehospitalisation during the 12-month follow-up was higher in the MR recurrence group when compared to the group without MR recurrence [13 out of 25 patients (52.0%) vs. 59 out of 195 patients (30.3%); *p* = 0.029] (Fig. 3b).

### Predictors of recurrent MR after PMVR in FMR

Patients’ clinical characteristics at baseline did not differ between patients with recurrent MR and patients without MR recurrence (Table 1). The patients in the group without MR recurrence were at median 77.0 [interquartile range (IQR) 71.0, 82.0] years old, the patients with recurrent MR were 73.0 (IQR 68.5, 79) years old (*p* = 0.179). Logistic EuroSCORE was 20.5 (IQR 13.0, 33.3) in the group without MR recurrence, respectively 25.0 (13.0, 38.8) in the MR recurrence group (*p* = 0.479).

**Table 1:** Patients’ characteristics of FMR patients without (−) MR recurrence and with (+) MR recurrence at 1 year follow-up after PMVR

	− MR recurrence (*n* = 195)	+ MR recurrence (*n* = 25)	*p* value
Clinical characteristics			
Age (years)	77.0 (71.0, 82.0)	73.0 (68.5, 79.0)	0.179
Female gender, *n* (%)	74 (37.9)	8 (32.0)	0.563
Logistic EuroSCORE (%)	20.5 (13.0, 33.3)	25.0 (13.0, 38.8)	0.479
ICM, *n* (%)	127 (65.1)	14 (56.0)	0.370
DCM, *n* (%)	58 (29.7)	10 (40.0)	0.296
Coronary artery disease, *n* (%)	131 (67.2)	14 (56.0)	0.267
Prior CABG, *n* (%)	55 (27.7)	10 (40.0)	0.227
Prior valve surgery, *n* (%)	30 (15.4)	5 (20.0)	0.553
Prior Stroke, *n* (%)	17 (8.7)	2 (8.0)	0.904
COPD, *n* (%)	38 (19.5)	4 (16.0)	0.676
Diabetes, *n* (%)	46 (23.6)	4 (16.0)	0.394
Atrial fibrillation, *n* (%)	132 (67.7)	18 (72.0)	0.663
Pacemaker/ICD/CRT, *n* (%)	137 (70.3)	16 (64.0)	0.522
Peripheral artery disease, *n* (%)	29 (14.9)	4 (16.0)	0.882
Severe tricuspid regurgitation, *n* (%)	48 (24.6)	8 (33.0)	0.429
Medication			
ACE inhibitors/ARB, *n* (%)	145 (74.4)	20 (80.0)	0.538
Betablocker, *n* (%)	154 (79.0)	21 (84.0)	0.558
Diuretics, *n* (%)	180 (92.3)	24 (96.0)	0.503
Aldosterone antagonists, *n* (%)	82 (42.1)	11 (44.0)	0.853
Digitalis, *n *(%)	23 (11.8)	2 (8.0)	0.574
Laboratory assessment			
Estimated GFR (ml/min)	48 (34, 64)	40 (31, 72)	0.766
NT-proBNP (× 1000 pg/ml	2.94 (1.44, 5.49)	3.05 (2.33, 5.93)	0.333
Haemoglobin (g/dl)	12.3 (11.1, 13.4)	12.0 (10.0 12.9)	0.127

Values are *n* (%) or median (interquartile range)

*FMR* functional mitral regurgitation, *MR* mitral regurgitation, *ICM* ischemic cardiomyopathy (impaired left ventricular function that results from coronary artery disease), *DCM* dilative cardiomyopathy (due to non-ischemic origin), *ACE* angiotensin-converting enzyme, *ARB* angiotensin receptor blocker, *CABG* coronary artery bypass grafting, *COPD* chronic obstructive pulmonary disease, *CRT* cardiac resynchronization therapy, *GFR* glomerular filtration rate, *ICD* internal cardiac defibrillator, *NT-proBNP* brain natriuretic peptide, *PMVR* Percutaneous mitral valve repair

*Indicates *p* ≤ 0.05 between the groups

MR recurrence was characterized by a higher left ventricular sphericity index [0.69 (IQR 0.64, 0.74) vs. 0.65 (IQR 0.58, 0.70), *p* = 0.003], a larger left atrium volume [118 (IQR 96, 143) ml vs. 102 (IQR 84, 123) ml, *p* = 0.019], a larger tenting height 10 (IQR 9, 13) mm vs. 8 (IQR 7, 11) mm (*p* = 0.047), and a larger mitral valve annulus [41 (IQR 38, 43) mm vs. 39 (IQR 36, 40) mm, *p* = 0.015] when compared to patients with stable long-term results (Table 2).

**Table 2:** Baseline echocardiographic parameters of FMR patients without (−) MR recurrence and with (+) MR recurrence at 1 year follow-up after PMVR

	− MR recurrence (*n* = 195)	+ MR recurrence (*n* = 25)	*p* value
Ejection fraction (%)	38 (30, 46)	33 (27, 45)	0.196
LVEDD (mm)	58 (51, 64)	62 (55, 69)	0.065
LV sphericity index	0.65 (0.58, 0.70)	0.69 (0.64, 0.74)	0.003*
Left atrium volume (ml)	102 (84, 123)	118 (96, 143)	0.019*
RVEDD (mm)	34 (29, 38)	34 (29, 43)	0.270
TAPSE (mm)	17 (15, 20)	16 (14, 20)	0.595
PASP (mmHg)	41 (31, 51)	38 (30, 50)	0.601
Interpapillary distance (mm)	21 (15, 27)	20 (18, 22)	0.717
Mitral valve annulus (mm)	39 (36, 40)	41 (38, 43)	0.015*
Tenting height (mm)	8 (7, 11)	10 (9, 13)	0.047*
Tenting area (cm^2^)	2.7 (1.8, 3.6)	3.0 (2.2, 3.8)	0.310
PML tethering angle (°)	38 (29, 45)	40 (25, 45)	0.762
AML tethering angle (°)	25 (15, 40)	28 (14, 45)	0.708
PML length (mm)	13 (10, 15)	14 (12, 16)	0.188
AML length (mm)	25 (22, 27)	27 (23, 29)	0.141
Preprocedural transmitral valve gradient (mmHg)	2.0 (1.1, 2.7)	1.7 (1.2, 2.4)	0.449
Regurgitation jet direction (central, eccentric) n/n	137/58	17/8	0.817
Vena contracta (mm)	6 (5, 8)	7 (6, 8)	0.938
EROA (cm^2^)	0.35 (0.25, 0.40)	0.35 (0.28, 0.38)	0.860
Regurgitation volume (ml)	52 (41, 60)	48 (39, 65)	0.860
Coaptation length (mm)	4 (4, 6)	5 (4, 5)	0.878
Number of implanted clips, *n*	1 (1, 2)	1 (1, 2)	0.865
Postprocedural transmitral valve gradient (mmHg)	3.1 (2.4, 4.0)	3.0 (2.0, 4.0)	0.904

Values are *n* (%) or median (interquartile range)

*FMR* functional mitral regurgitation, *MR* mitral regurgitation, *AML* anterior mitral valve leaflet, *EROA* effective regurgitation orifice area, *LV* left ventricular, *LVEDD* left ventricular diastolic diameter, *PASP* pulmonary artery systolic pressure, *PML* posterior mitral valve leaflet, *PMVR* Percutaneous mitral valve repair, *RVEDD* right ventricle end-diastolic diameter, *TAPSE* tricuspid annular plane systolic excursion

*Indicates *p* ≤ 0.05 between the groups

In the univariate and multivariate regression model, the left ventricular sphericity index [Odds Ratio (OR) 1.120 95% Confidence Interval (CI) 1.039–1.413, *p* = 0.003], tenting height (OR 1.207, 95% CI 1.031–1.413, *p* = 0.019), and left atrium enlargement (OR 1.018, 95% CI 1.000–1.038, *p* = 0.047) affected MR recurrence after PMVR (Table 3). Using a cut-off value of 0.70 for the sphericity index, the sensitivity to predict MR recurrence after PMVR was 40% with a specificity of 85% (AUC 0.678; Youden Index 0.25). For the tenting height, a cut-off value of 10.5 mm (sensitivity 44%; specificity 73%; AUC 0.655; Youden Index 0.17), and for the left atrium enlargement, a cut-off value of 125 ml (sensitivity 40%; specificity 78%; AUC 0.631; Youden Index 0.18) were calculated.

**Table 3:** Regression analysis for MR recurrence after PMVR

	Univariate			Multivariate		
	OR	95% CI	*p* value	OR	95% CI	*p* value
Baseline variable						
Age (years)	0.975	0.939–1.012	0.181			
Atrial fibrillation	2.390	0.787–7.261	0.124			
ICM	0.680	0.268–1.722	0.416			
DCM	1.249	0.469–3.327	0.656			
Ejection fraction (%)	0.975	0.939–1.013	0.196			
LVEDD (mm)	1.039	0.997–1.083	0.068*	1.035	0.988–1.085	0.151
LV sphericity index	1.105	1.032–1.1183	0.004*	1.120	1.039–1.208	0.003*
Left atrium size (ml)	1.017	1.003–1.032	0.021*	1.018	1.000–1.085	0.047*
Interpapillary distance (mm)	0.989	0.931–1.050	0.715			
Tenting height (mm)	1.144	1.000–1.309	0.050*	1.207	1.031–1.413	0.019*
Tenting area (cm^2^)	1.208	0.839–1.740	0.309			
Coaptation length (mm)	1.086	0.750–1.571	0.664			
Coaptation hight (mm)	0.910	0.796–1.040	0.167			
Mitral valve annulus (mm)	1.150	1.029–1.348	0.017*	1.110	0.961–1.281	0.155
Eccentric jet direction	1.104	0.525–1.502	0.621			
PML Tethering angle (°)	0.995	0.961–1.029	0.761			
AML Tethering angle (°)	1.006	0.973–1.040	0.707			
PML length (mm)	1.092	0.958–1.245	0.189			
AML length (mm)	1.083	0.974–1.204	0.143			
EROA (cm^2^)	1.472	0.792–1.331	0.859			
RV (ml)	1.002	0.972–1.033	0.877			
PASP (mmHg)	0.991	0.956–1.026	0.598			
TAPSE (mm)	0.968	0.858–1.091	0.592			
GFR (ml/min)	1.003	0.984–1.022	0.764			
Haemoglobin (g/dl)	0.836	0.664–1.053	0.836			
NT-proBNP (× 1000 pg/ml)	1.026	0.973–1.082	0.337			

*MR* mitral regurgitation, *AML* anterior mitral valve leaflet, *CI* confidence interval, *DCM* dilated cardiomyopathy, *ICM* ischemic cardiomyopathy, *EROA* effective regurgitation orifice area, *GFR* glomerular filtration rate, *OR* Odds ratio, *PASP* pulmonary artery systolic pressure, *PML* posterior mitral valve leaflet, *PMVR* Percutaneous mitral valve repair, RV regurgitation volume, *TAPSE* tricuspid annular plane systolic excursion

*Indicates *p* ≤ 0.05

## Discussion

Our major findings are as follows: (1) PMVR could be performed successfully in the majority of the patients with FMR but recurrence of MR after 1 year occurred in 11.4% of the cases; (2) MR recurrence after PMVR was associated with a higher rehospitalisation rate and less improvement in NYHA functional class; and (3) parameters of advanced heart failure such as a spherical left ventricle, elevated tenting height, and left atrial enlargement predicted MR recurrence.

### Efficacy of PMVR in patients with FMR

In the present study, PMVR was effective in reducing MR, and MR grade 2 + or less were observed in 91.2% of the patients at discharge. Accordingly, clinical symptoms improved during the 12 month post PMVR. These findings are consistent with those from previous studies that demonstrated similar MR reduction and clinical improvement after one year in patients with FMR [7, 8, 13, 14].

However, even when optimal results are obtained acutely after PMVR, MR may recur during follow-up in a significant proportion of patients. After initial successful PMVR, we observed that 11.4% of the patients had recurrent MR grade 3 + or worse after one year. These rates of recurrence were also observed in previous studies [13–16]. In general, failure of PMVR and worsening of MR was the most important predictor for the outcome and associated with a poor prognosis [5, 6, 17]. In addition, we here demonstrate that patients with recurrent MR after PMVR had a higher rehospitalisation rate and less improvement of dyspnea than patients without MR recurrence.

### Predictors of MR recurrence after PMVR

In the present study, patients with recurrent MR after PMVR were characterized by a pre-procedural high left ventricular sphericity index and left atrium enlargement. A spherical-shaped left ventricle may reflect an advanced state of heart failure with progressive changes in ventricular geometry and reduced capability of reverse remodeling. Irrespective of MR, unbalanced left ventricular geometry and shape have been associated with cardiovascular events and heart failure [18, 19]. In a previous study, a high sphericity index predicted an increased incidence of heart failure in the general population [20]. The impact of ventricular geometry on outcome in PMVR patients was investigated in a previous study in which patients with dilated left ventricles had a higher risk of all-cause death and rehospitalisation for heart failure [21]. It remains unclear whether the worse clinical outcome in these patients was affected by the more advanced state of heart failure or can at least partially be explained by the recurrence of MR. Patients with larger left ventricular end-diastolic volume and proportional MR grades (MR severity is proportional to the amount of left ventricular dilatation) might not benefit from PMVR in term of survival. These patients were more often seen in the MITRA-FR trial [7], whereas the COAPT trial mainly included patients with smaller left ventricular end-diastolic volume and disproportionately high MR grades [8].

For surgical mitral valve repair, a higher sphericity index was also found to be associated with the recurrence of MR [4]. Interestingly, in patients with FMR undergoing surgical mitral valve repair a sphericity index > 0.7 was found to be a predictor for recurrence of MR [22]. In our study, we identified an equal cut-off value for patients undergoing PMVR underlining the predictive role of disordered ventricular geometry for both interventional and surgical approaches to FMR therapy. In more spherical-shaped ventricles, the papillary muscles are displaced which can lead to restriction of the leaflets and derangement of the normal chordal leaflet alignment [23]. Previously it has been shown that papillary muscle distance is important in the development and recurrence of FMR [24].

In the study of Stolfo et al. early device failure was predicted by enlarged mitral annulus diameter [25]. In a recent study assessing long-term reduction of MR after PMVR in patients with FMR, a restrictive posterior leaflet motion, the presence of asymmetric leaflet tethering and pre-procedural pulmonary hypertension were identified as independent predictors of MR recurrence [15]. As a bystander together with structural changes of advanced heart failure, PMVR at that stage might not be sufficient to reverse remodeling, resulting in the recurrence of MR and a lack of effect on the progression of heart failure.

Left atrium enlargement is basically a marker of volume overload and increased filling pressures in FMR [26]. In patients with heart failure in general, and in heart failure patients undergoing PMVR left atrium enlargement is a strong and independent predictor of mortality and disease progression [27, 28]. The left atrium plays a central role in patients with chronic MR since the regurgitant volume leads to negative left atrial remodeling and decline in left atrial function [29]. In addition, left atrium enlargement might be considered as a marker of left ventricle diastolic dysfunction.

Taken together, our results demonstrate that parameters of advanced heart failure such as a spherical left ventricle, elevated tenting height and left atrium enlargement were associated with MR recurrence after PMVR. Alternative interventional approaches such as transseptal direct annuloplasty devices have been shown to be safe and effective in patients with FMR [30]. However, long-term follow-up is still needed to prove stability and efficacy in MR reduction and symptomatic improvement in these patients.

Our study has several limitations. First, it was a single-center analysis. The results should therefore be confirmed in additional prospective multicenter trials with a larger patient cohort. Second, the follow-up time was one year after procedure, which might have been too short to identify the impact of other parameters for long-term PMVR results.

## Conclusion

Although PMVR in patients with FMR is effective and leads to clinical improvement in the majority of the patients, recurrence of MR can occur. Parameters associated with advanced heart failure at baseline such as a spherical left ventricle and left atrium enlargement may have an impact on long-term results after PMVR.
